# Longitudinal stability of urinary extracellular vesicle protein patterns within and between individuals

**DOI:** 10.1038/s41598-021-95082-8

**Published:** 2021-08-02

**Authors:** Leyla A. Erozenci, Sander R. Piersma, Thang V. Pham, Irene V. Bijnsdorp, Connie R. Jimenez

**Affiliations:** 1 Department of Medical Oncology, OncoProteomics Laboratory, Cancer Center Amsterdam, Amsterdam UMC, Location VUMC, Amsterdam, The Netherlands; 2 Department of Urology, Amsterdam UMC, Location VUMC, Amsterdam, The Netherlands

**Keywords:** Cancer, Biomarkers, Oncology

## Abstract

The protein content of urinary extracellular vesicles (EVs) is considered to be an attractive non-invasive biomarker source. However, little is known about the consistency and variability of urinary EV proteins within and between individuals over a longer time-period. Here, we evaluated the stability of the urinary EV proteomes of 8 healthy individuals at 9 timepoints over 6 months using data-independent-acquisition mass spectrometry. The 1802 identified proteins had a high correlation amongst all samples, with 40% of the proteome detected in every sample and 90% detected in more than 1 individual at all timepoints. Unsupervised analysis of top 10% most variable proteins yielded person-specific profiles. The core EV-protein-interaction network of 516 proteins detected in all measured samples revealed sub-clusters involved in the biological processes of G-protein signaling, cytoskeletal transport, cellular energy metabolism and immunity. Furthermore, gender-specific expression patterns were detected in the urinary EV proteome. Our findings indicate that the urinary EV proteome is stable in longitudinal samples of healthy subjects over a prolonged time-period, further underscoring its potential for reliable non-invasive diagnostic/prognostic biomarkers.

## Introduction

Urine is a biofluid that has raised great interest as a biomarker source for the detection of disease, since it can be collected in large volumes, in a non-invasive manner. Extracellular vesicles (EVs) are small vesicles that are secreted by most cell types of the human body into biofluids, including blood and urine^1^. Circulating EVs are biomarker-rich organelles as they, at least in part, represent their cell-of-origin^2^. Importantly their molecular cargo (DNA, RNA, miRNA, protein) is protected by the EV bilayer membrane from degradation by enzymes present in biofluids^3^. Advances in mass spectrometry-based proteomics over the past decade have allowed for in-depth analysis of proteins in clinical samples, including urinary EVs^4,5^. The proteome cargo of urinary EVs has been the subject of several biomarker studies^6^. Previous studies on the whole urinary proteome reported a high level of variation within individuals over time (intra-individual variation), as well as between individuals (inter-individual variation)^7–12^; which hampers biomarker applications. On the contrary, it was reported that the urinary EV proteome of few donors was stable in a short time period^13,14^. The stability of urinary EV proteome was never investigated in a comprehensive manner with multiple individuals and longitudinal sampling over an extended period of time.

**Figure 1:**
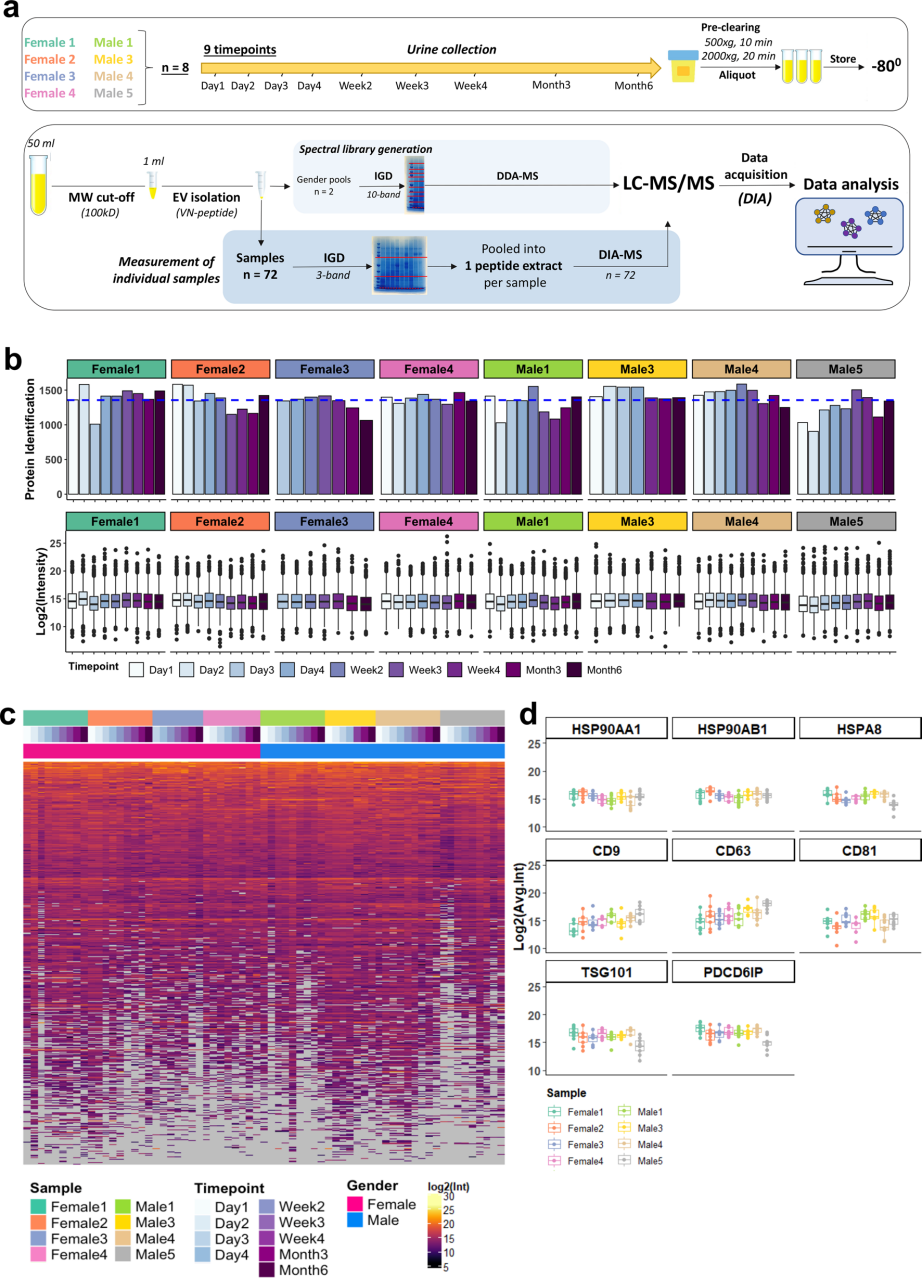
Schematic workflow of the study and overview of the whole urinary EV proteome. (**a**) Schematic diagram of the collection of urine samples on the upper panel, and DIA-MS workflow on the lower panel. Urine was collected from 8 individuals at 9 timepoints over the course of 6 months (total 72 samples) and was pre-cleared from dead and apoptotic cells using centrifugation and stored at − 80 °C. 50 ml urine per donor was concentrated to 1 ml with ultrafiltration (100-kDa cut-off) and urinary EVs were subsequently isolated using the Vn96-peptide-affinity kit^20^. For the high-depth spectral library generation, gender-specific urinary EV pools (n = 2) were subjected to 10-band gel fractionation followed by DDA-MS. Individual samples (n = 72) were measured in single shot DIA-MS mode, followed by data extraction and quantification using intensities, and extensive data analyses. (**b**) Total number of proteins identified (upper panel) per individual sample and distribution of normalized protein intensities (lower panel) for each sample (n = 67), showing a highly similar protein identification number between the samples and individuals. (**c**) Data presence plot, showing a high data presence amongst all samples. The expression levels of the total urinary EV proteome (1802 proteins) is indicated amongst all samples (n = 67). The proteins were ranked according to data presence and average log2-intensity. The missing values (29,913 out of total 120,734 data points) are gray. (**d**) Expression levels of selected EV-related protein markers for each sample with Heat-shock proteins (upper panel), tetraspanins (middle panel), and TSG101 and PDCD6IP (lower panel) showing a good consistency in the level of these proteins in time.

In the present study, we assessed the stability of the urinary EV proteome using a highly reproducible next generation proteomics approach based on liquid chromatography on-line coupled to data-independent acquisition (DIA)-MS^15–17^. This mass spectrometry approach combines the benefits of global discovery proteomics with the quantitative precision of targeted mass spectrometry with dynamic range over 3 order of magnitude^17,18^, and hence provides a pathway for large-scale clinical proteomics^19^. To this end, we measured a longitudinal cohort of eight different healthy subjects (four females, four males) of whom urine samples were collected at 9 different timepoints over 6 months (see Fig. 1a for a schematic overview). The stability of the urinary EV proteome over time, as well as the consistency between individuals was examined. Moreover, biological functions and core expression networks of EV proteins identified in all subjects were investigated, and gender-specific processes were explored.

## Materials and methods

### Urine collection and informed consent

The study was approved by the Amsterdam University Medical Center (Amsterdam UMC, Location VUMC) local Medical Ethical Committee (METC reference number #2018.657). Urine was collected after a signed informed consent was obtained from each participant. All methods and experiments were performed in accordance with the relevant guidelines and regulations, which is in accordance with the Declaration of Helsinki. The workflow for collecting and processing urine samples is depicted in Fig. 1a. Urine (50–150 mL) was collected and aliquoted into sterile polypropylene tubes and centrifuged for 10 min at 500 × g at 4 °C. Subsequently the supernatant was centrifuged for 20 min at 2000 × g at 4 °C and divided in 50 mL aliquots which were immediately frozen at − 80 °C until processing for proteomics.

### Urinary EV isolation

Urinary EVs isolation was performed as previously reported using the Vn96 peptide capture method that precipitates EVs via binding to HSPs at the EV surface^20–22^. 50 mL of urine for each sample was thawed O/N at 4 °C. Briefly, urine samples were centrifuged at 2000 × g for 20 min to remove THP-polymers from the urine that have been formed by cold conditions. Subsequently, 0.05% Nonidet P40 Substitute (NP40, Sigma- Aldrich, Zwijndrecht, The Netherlands) was added to the urine. 50 mL urine per sample was concentrated to 1 ml using a 100 kDa cutoff filters (Amicon Ultra, Millipore, Amsterdam, The Netherlands). To prevent degradation by proteases, protease inhibitor cocktail was added (PIC, #10,276,200, Roche). To remove small debris and large protein complexes, urine was centrifuged at 16,000 × g for 15 min at 4 °C in a tabletop centrifuge. Subsequently 40 µl of the VN-96 peptide (Microvesicle Enrichment kit, New England Peptide, #W1073-2, USA) was added, and incubated on a rotation wheel for 1 h at RT. EVs were isolated after centrifugation for 15 min at 16,000 × g. The pellet was washed with PBS and centrifuged again for 15 min at 16,000 × g to obtain the final sample. All the centrifugation steps were performed at 4 °C. The urinary-EV pellets were dissolved in LDS sample buffer (containing 10% Dithiothreitol, Life Technologies, Carlsbad, CA, cat No:NP0008) for proteomics experiments.

### Spectral library generation and DDA-LC-MS/MS measurement

For library generation for DIA-measurement, aliquots were taken from all samples to make two pools, one consisting of samples from males and one from females that were used as input for EV isolation. The pooled EV samples were loaded on gradient gels from Invitrogen (NuPAGE 4–12% Bis–Tris gel, 1 mm × 10 wells). The gels were stained with Coomassie brilliant blue G-250 (Pierce, Rockford, IL) and in-gel digested as previously described^23^. In brief, gels were washed twice in 50 mM ammonium bicarbonate (ABC) and dehydrated twice in 50 mM ABC/50% acetonitrile (ACN). Cysteine bonds were reduced by incubation with 10 mM DTT/50 mM ABC at 56 °C for 1 h and alkylated with 50 mM iodoacetamide/50 mM ABC at room temperature (RT) for 45 min. After washing sequentially with ABC and ABC/50% ACN, the whole gel was sliced in 10 bands for each lane. Gel parts were sliced into cubes of 1mm3, which were incubated overnight with 6.25 ng/mL trypsin (Promega, sequence grade V5111). Peptides were extracted once in 1% formic acid and twice in 5% formic acid/50% ACN. Subsequently, the extract volume was reduced to 50 µL in a vacuum centrifuge. The sample was filtered using a 0.45 µm filter to remove gel particles and contaminants prior to LC–MS analysis.

Extracted peptides were separated on a 75 µm × 42 cm custom packed Reprosil C18 aqua column (1.9 µm, 120 Å) in a 90 min. gradient (2–32% Acetonitrile + 0.5% Acetic acid at 300 nl/min) using a U3000 RSLC high pressure nanoLC (Dionex). Eluting peptides were measured on-line by a Q Exactive mass spectrometer (Thermo Fisher, Bremen, Germany) operating in data-dependent acquisition mode. Intact peptide ions were detected at a resolution of 70,000 (at m/z 200) and fragment ions at a resolution of 17,500 (at m/z 200); the MS mass range was 350–1,400 Da. AGC Target settings for MS were 3E6 charges and for MS/MS 1E6 charges. Peptides were selected for Higher-energy C-trap dissociation fragmentation at an underfill ratio of 1% and a quadrupole isolation window of 1.6 Da, peptides were fragmented at a normalized collision energy of 28.

### Processing of individual samples for DIA-LC-MS/MS

For proteomics analysis of individual urine EV samples, each sample was loaded on gradient gels (Invitrogen, NuPAGE 4–12% Bis–Tris gel, 1 mm × 10 wells), similar as described above. The gels were stained with Coomassie brilliant blue G-250 (Pierce, Rockford, IL), reduced by 10 mM DTT/50 mM ABC at 56 °C for 1 h and alkylated with 50 mM iodoacetamide/50 mM ABC at room temperature (RT) for 45 min. After washing sequentially with ABC and ABC/50% ACN, the whole gel lanes were sliced in 3 bands per sample. Gel parts were sliced into cubes of 1 mm^3^, which were incubated overnight with 6.25 ng/mL trypsin (Promega, sequence grade V5111). Peptides were extracted once in 1% formic acid and twice in 5% formic acid/50% ACN. The extracts of three fractions were pooled per biological sample. Volume was reduced to 100 µl in a vacuum centrifuge to evaporate acetonitrile and samples were desalted using a 10 mg OASIS HLB column (Waters, Milford). after washing in 0.1% TFA. Samples were eluted in 80% ACN/0.1% TFA and were dried in a vacuum centrifuge. Peptides were redissolved in 20 µl loading solvent (4% ACN in 0.5% TFA) for LC-MS analysis.

### DIA-LC-MS/MS measurement

Peptides were separated by an Ultimate 3000 nanoLC system (Dionex LC-Packings, Amsterdam, The Netherlands), equipped with a 50 cm × 75 µm ID nanoViper fused silica column packed with 1.9 µm 120 Å Pepmap Acclaim C18 particles (Thermo Fisher, Bremen, Germany). After injection, peptides were trapped at 3 μl/min on a 10 mm × 100 μm ID trap column packed with 3 μm 120 Å Pepmap Acclaim C18 at 0% buffer B (buffer A: 0.1% formic acid in ultrapure water; buffer B: 80% ACN + 0.1% formic acid in ultrapure water) and separated at 300 nl/min in a curved 10–52% buffer B gradient in 120 min (140 min inject-to-inject). Eluting peptides were ionized at a potential of + 2 kVa into a Q Exactive mass spectrometer (Thermo Fisher, Bremen, Germany). Data was measured using a data-independent acquisition (DIA) protocol. The DIA-MS method consisted of an MS1 scan from 350 to 1400 m/z at 120,000 resolution (AGC target of 3E6 and 60 ms injection time). For MS2, 24 variable size DIA segments were acquired at 30,000 resolution (AGC target 3E6 and auto for injection time). The DIA-MS method starting at 350 m/z included one window of 35 m/z, 20 windows of 25 m/z, 2 windows of 60 m/z and one window of 418 m/z, which ended at 1400 m/z. Normalized collision energy was set at 28. The spectra were recorded in centroid mode with a default charge of 3 + and a first mass of 200 m/z.

### Raw data processing

DDA Raw files were processed using MaxQuant 1.6.4.0. MS/MS spectra were searched against a Swissprot FASTA file downloaded Feb 2019. The precursor and fragment mass tolerance were set to 4.5 and 20 p.p.m., respectively. Peptides with minimum of seven amino-acid length were considered with both the peptides and proteins filtered to a false discovery rate (FDR) of 1%. Enzyme specificity was set to trypsin and up to two missed cleavages were allowed. Cysteine carbamidomethylation was searched as a fixed modification, whereas protein N-terminal acetylation and methionine oxidation were searched as variable modifications. DIA raw files were searched in Spectronaut version 13.10 (Biognosys, Schlieren, Switzerland) with default settings. The MaxQuant msms.txt file from the DDA search result was imported into Spectronaut to generate a project-specific spectral library. Modifications were the same as for the MaxQuant DDA search. The search result was exported at the fragment ion level for MaxLFQ protein quantification^24^. The mass spectrometry proteomics data have been deposited to the ProteomeXchange Consortium via the PRIDE^25,26^ partner repository with the dataset identifier PXD022983.

### Data analysis

All analysis was done using log2-transformed intensities in R version 4.0.2 (https://www.R-project.org/). All the clustering analyses were performed using R package ComplexHeatmaps version 2.5.5^27^. For the data presence heatmap, proteins ordered on the number of identified data points (high-to-low) and average log2-normalized intensity (high-to-low) over the 67 individual samples was plotted. For intra- and inter-individual sample clustering, the Spearman correlation of the normalized intensities was calculated for whole urinary EV proteome per sample, and the correlation coefficient was plotted. For protein clustering of top 10% most variable proteome, the protein abundancies were normalized to zero mean and unit variance for each individual protein. Subsequently, the Spearman distance measure was used for clustering. R package ggplot2 version 3.3.2 (https://ggplot2.tidyverse.org) was used for all plots other than heatmaps and networks. Intra-individual CV was calculated amongst all timepoints per person; while inter-individual coefficient of variation (CV) was calculated amongst all 67 individual samples. Gene Ontology (GO) term analyses of the core urinary EV proteome were performed using ClueGO version 2.5.7^28^. Redundant GO terms were manually collapsed into one parent term and were presented in the GO barplots. Protein networks for the core urinary EV proteome were created using STRING version 11^29^ and visualization was further modified using Cytoscape version 3.8.0^30^. Significantly connected protein clusters were extracted using ClusterONE version 1.0^31^, and further annotated in detail for implicated biological processes using BINGO version 3.0.3^32^. Differential statistical analysis to compare the protein expression between female and male samples was performed using R package Limma version 3.45.14^33^. Gene Set Enrichment Analysis (GSEA) was performed using R package fgsea version 1.15.2^34^.

## Results

### Longitudinal urinary EV proteome profiling in healthy subjects

To investigate the temporal stability of the urinary EV proteome within and between individuals, as well as its biology, we collected urine from 8 healthy individuals (4 males and 4 females) at 9 timepoints spanning 6 months (see Fig. 1a for a schematic overview). Urinary EVs were isolated using the Vn96 peptide capture method that enables reproducible high-throughput profiling^20–22^. The characterization and validity of VN96 peptide-based EV capture method has been addressed in previous studies for cell culture supernatants, blood and urine^20–22,35–37^ [Erozenci et al. 2021, submitted manuscript 2, Submission ID a4f6d1e6-ce2f.-483a-927b-3da8b2083095]. These studies showed that the EV fraction captured is in the size range (30 to 100 nm), enriched for exosome markers and is largely comparable to EVs isolated by ultracentrifugation. For urinary EV profiling by DIA-MS, we generated a project-specific spectral library using two gender-specific pools of urinary EVs. These pools were analyzed by in-depth proteomics based on 10 band gel-fractionated samples coupled to shot gun proteomics (DDA-MS) (Fig. 1a). The final spectral library consisted of 3166 proteins. Subsequently, all 72 individual samples were measured using single-shot DIA-MS (Fig. 1a). The total urinary EV proteome consisted of 1802 proteins. Five samples were excluded from further downstream analysis, because the number of identified proteins was below 2 standard deviations of the mean (indicated in Supplementary Fig. 1). The whole urinary EV proteome of 1802 proteins were identified with a mean of ~ 1355 proteins per urinary EV sample (ranging between 905 and 1587) and with > 75% of data points present across all 67 samples (Fig. 1b,c). Selected common exosome markers such as tetraspanins (CD9, CD63, CD81), TSG101, PDCD6IP (ALIX) and heat-shock proteins (HSPs) (HSP90AA1, HPS90AB1, HSPA8) were detected in all individuals at almost all timepoints at median-to-high abundance (Fig. 1d). Closer inspection of these EV markers revealed that day-to-day variation within the same donor was low, especially for HSPs (median CV = 0.58), TSG101 and PDCP6IP (CV 0.74 and 0.70) and showed comparable abundance levels in different individuals (Fig. 1d)*.* The level of CD9, CD63 and CD81 exosome markers was more variable compared to the HSPs, most notably between different individuals (median CV = 1.15) (Fig. 1d), with donor “Male5” showing the highest expression, suggesting that inter-individual differences in EV subpopulations might be present in the urine.

### Highly stable personal urinary EV proteomes with larger inter-individual variation

**Figure 2:**
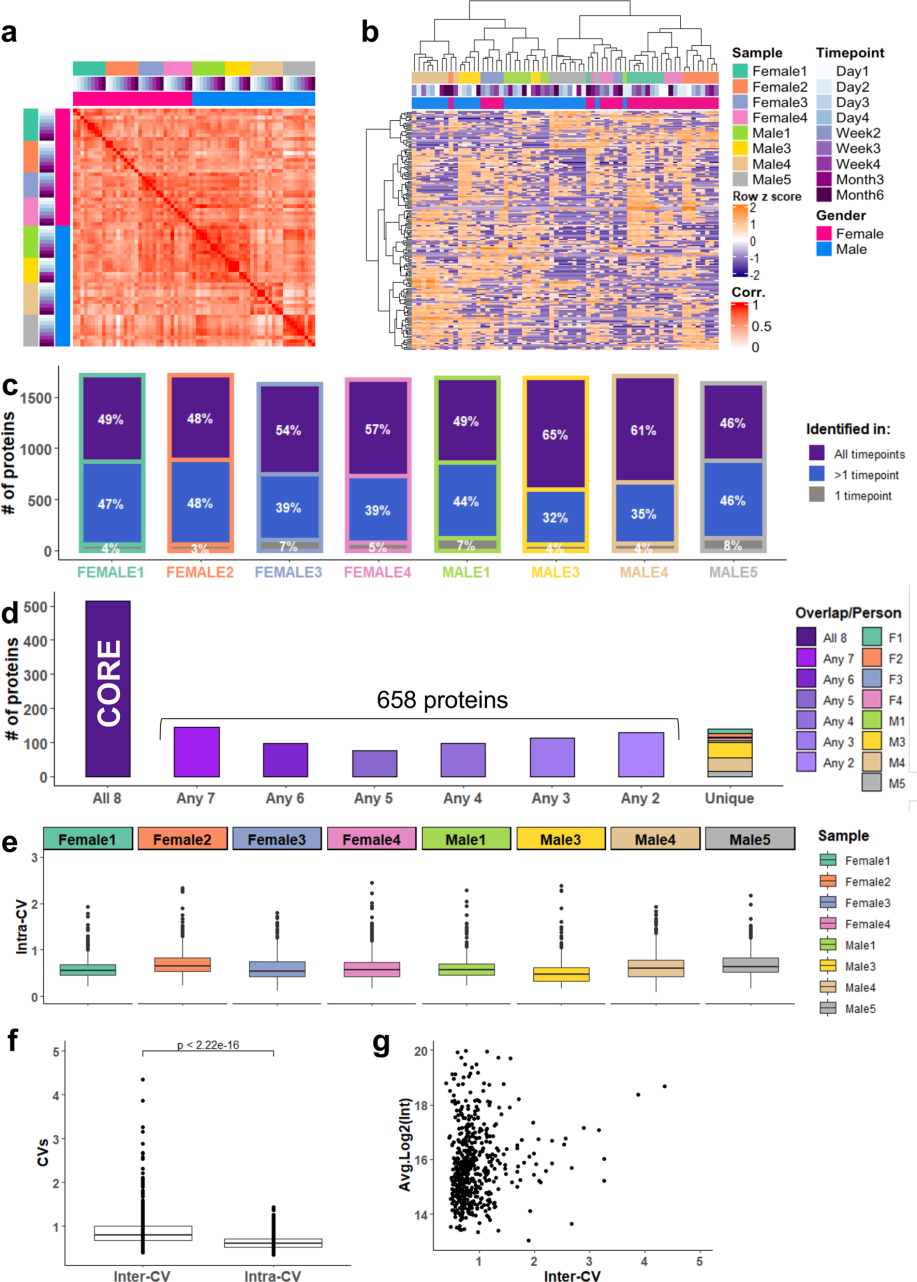
Personal and inter-individual variation of the urinary EV proteome. (**a**) Unsupervised spearman correlation analysis of 8 individuals (67 samples) based on total proteome (1802 proteins) with the correlation coefficient values from 0 (white) to 1 (red). (**b**) Hierarchical clustering of the top 10% most variable urinary EV proteins (180 proteins) showing a clustering largely based on individual. (**c**) Number of proteins identified at all timepoints (purple), at more than 1 timepoint (blue) or at a single timepoint (gray) per donor. Percentages relative to the total proteins identified per person are annotated on the barplots. (**d**) Number of proteins identified in all individuals (core proteome, 516 proteins), in more than 1 individual (total 1174 proteins), and unique proteins per person (combined 113 proteins). (**e**) Distribution of personal CVs (intra-CVs) of each individual for the core urinary EV proteome (516 proteins) which was defined as the proteins detected at all timepoints in all 8 individuals. (**f**) Distribution of inter- and intra-CVs for the core urinary EV proteome (516 proteins). Inter-CV is calculated amongst 67 samples, and intra-CV is represented as the mean of the personal CVs of 8 individuals. (**g**) Distribution of inter-CVs in relation to protein abundance for the core urinary proteome (516 proteins), showing that the CV is independent from abundance.

To investigate the intra- and inter-individual stability of urinary EV proteome, an unsupervised correlation analysis was performed on the whole urinary EV proteome of 1802 proteins (Fig. 2a). Unsurprisingly, the highest correlations were observed within individuals, even over a longer period of time (6 months) (average intra-individual r = 0.77) (Fig. 2a), indicating a high stability of personal urinary EV proteomes. The correlation of protein profiles between individuals was lower than within individuals (average inter-individual r = 0.54) (Fig. 2a), indicating that each individual has their own level of abundance of the proteins that are present in urinary EVs. The lowest correlation was observed between donor “Male 5” to the other individuals (minimum r = 0.38), which also exhibited slightly different levels of EV markers as compared to the other subjects (Fig. 1d).

To investigate the potential effect of protein variation in normal urinary EV proteome, we focused on the most variable and most stable proteins. The protein variation was calculated on the total urinary EV proteome (1802 proteins) amongst all data points of 8 individuals. Unsupervised hierarchical cluster analysis of the top 10% most variable proteins separated the samples mostly related to individual protein profiles (Fig. 2b). This indicates that the most variable proteins determined the majority of the personal urinary EV proteomes (Fig. 2b). Importantly, 37 of the top 100 ExoCarta^38^ exosome-associated proteins were identified in the top 10% most stable proteome of urinary EVs; including HSPs, multiple RAB proteins, ACTB, ANXA2, and several members of the 14–3-3 protein family, indicating the stability of the proteins within the urinary EVs over time and between individuals. Examples for the stable Exocarta^38^ proteins, the top 15 most stable proteins and the top 15 most variable proteins are provided in Supplementary Fig. 2a–c.

The composition of the personal urinary EV proteomes were found to be highly similar in time with > 90% of the whole urinary EV proteome of 1802 proteins present in more than 1 timepoint per person (Fig. 2c). Furthermore, of the 1314 protein groups (i.e. excluding the protein isoforms), only a small number of proteins were unique to one individual, ranging from 3 to 45 proteins, with 90% of the identified proteome (1174 proteins) overlapping in any 2 or more individuals (Fig. 2d*,* Supplementary Fig. 3); revealing that the composition of urinary EV proteome is similar and comparable between different individuals. To examine EV consistency within and between individuals, we analyzed the core urinary EV proteome of our dataset, defined as the 516 proteins that were common to all 8 individuals at all timepoints measured (Fig. 2d*,* Supplementary Fig. 3). No difference was observed between the day-to-day variation of the donors (Fig. 2e). The median CV of the core proteome of 8 individuals over the 6-month period was 0.604 (ranging from 0.34 to 1.43) (Fig. 2f). The donor-donor variation was significantly higher than the day-to-day variation of the proteome with a median of 0.79 (ranging from 0.402 to 4.35; p < 2.2e−16) (Fig. 2f). However, this larger donor-donor variation was mostly based on a small subset of ‘outlier’ proteins that also had a high intra-individual CV (which represents less than 20% of the core proteome). Hence, most of the proteins within the urinary EVs are highly stable between days over a long period of time and also between different individuals. No relation between protein intensity and variation was observed, indicating that high abundant proteins do not necessarily have the lowest CV or vice versa, which is in agreement to previous observations reporting about variation of the entire human urinary proteome using DDA-MS (Fig. 2g)^7,12^.

Together, this analysis shows that the majority of the urinary EV proteome is stable and highly comparable within and between individuals.

### Biological functions of the core urinary EV proteome

**Figure 3:**
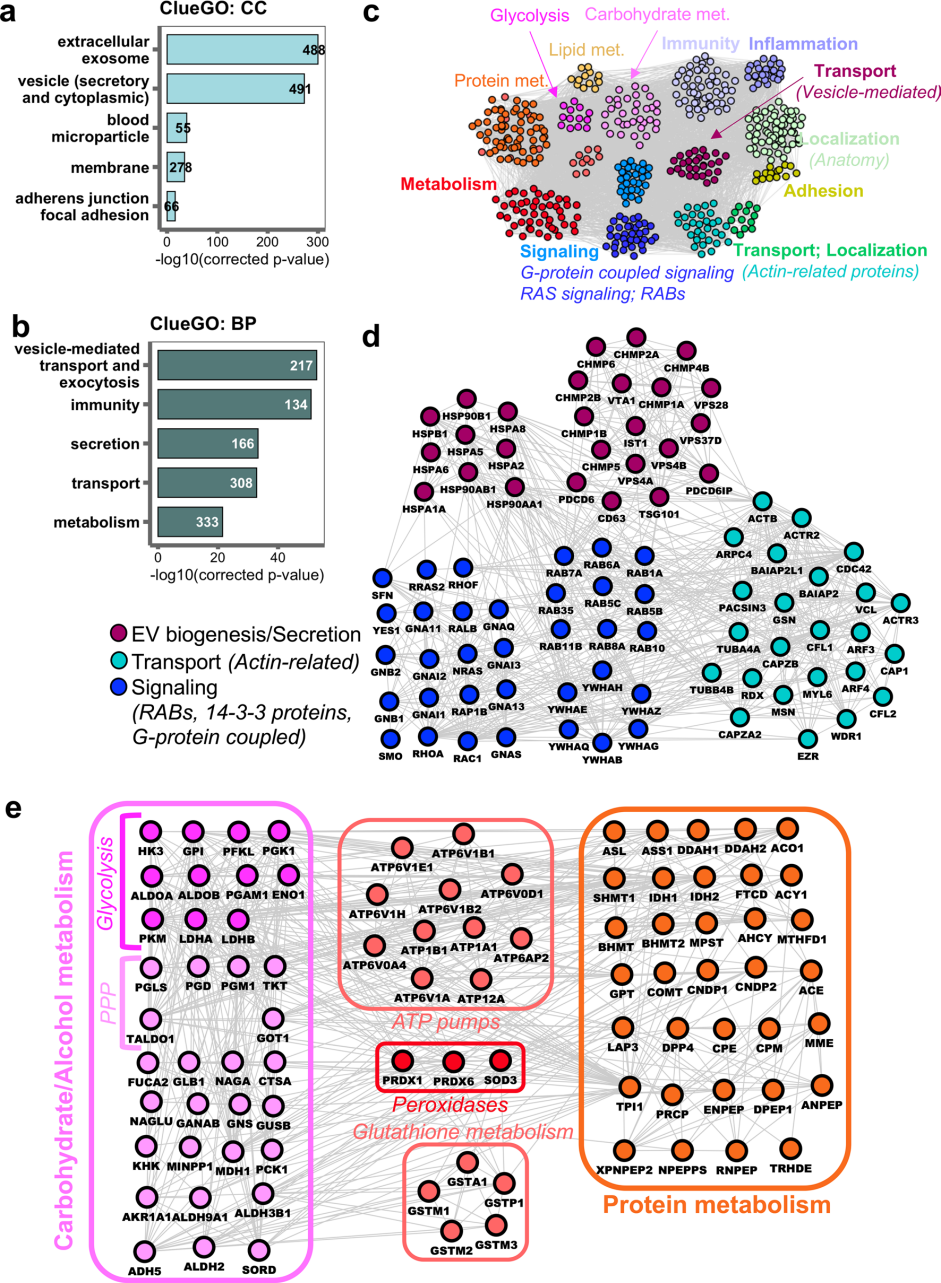
Enriched biological functions in the core urinary EV proteome. (**a**) Gene ontology (GO) term cellular component (CC) analysis of the core urinary EV proteome. Barplots show top 5 GO:CC terms associated with the core urinary EV proteome, analyzed using ClueGO^28^. (**b**) Gene ontology (GO) term biological process (BP) analysis of the core urinary EV proteome. Barplots show top 5 GO:BP terms associated with the core urinary EV proteome, analyzed using ClueGO^28^. (**c**) Schematic overview of the enriched biological functions in the core urinary EV protein network (516 proteins), analyzed by clusterONE^31^ and BINGO^32^. For the detailed network, see Supplementary Fig. 4. (**d**) Protein interaction network of EV markers and associated signaling and localization protein clusters, predicted by clusterONE^31^. (**e**) Protein interaction network of metabolic cluster. Enriched sub-networks predicted by clusterONE^31^ are indicated within the figure. *PPP: Pentose-Phosphate Pathway*.

To investigate the functions of urinary EV proteins, we focused on the proteins that were consistently identified in all individuals (the core urinary EV proteome, Fig. 2d*,* Supplementary Fig. 3). Gene ontology (GO) analysis showed that almost all detected proteins were associated with the cellular component term extracellular exosome and vesicle, underlining the EV nature of our samples (Fig. 3a). In addition, the majority of the core urinary EV proteins were found to be involved in biological processes that are vesicle-related such as vesicle-mediated transport and exocytosis (Fig. 3b).

In order to have a deeper understanding of the implicated general GO terms, we annotated in detail the biological pathways as well as functional protein clusters on the core urinary EV proteome. Cluster analysis using ClusterONE^31^ identified significantly connected protein complexes including EV-, immune- and metabolism-related proteins within the large urinary EV protein–protein interaction network (Fig. 3c; *for details see* Supplementary Fig. 4). A vesicle-linked cluster containing EV-related proteins such as ALIX, TSG101, HSPs, RABs and members of ESCRT-III (endosomal sorting complex required for transport III) was one of the significantly enriched subnetworks (p = 2.540E−4) in urinary EVs (Fig. 3d); indicating that urinary EVs contain several members of the EV biogenesis machinery. In addition, proteins involved in the regulation of cytoskeleton organization and signal transduction, in particular small GTPase-mediated and RAS signaling, were connected with the EV-associated network cluster (Fig. 3d), suggesting the presence of a signal transduction network in the core urinary EV proteome intertwined with the vesicle-linked protein cluster.

Other significantly enriched protein network clusters in the core urinary EV proteome were related to immunity (p = 3.834E−8) and metabolism (p = 6.738E−5). The immune subnetwork included proteins involved in innate and acute inflammatory response, complement factors, and immunoglobulin family of proteins among others (Supplementary Fig. 4), suggesting that urinary vesicles may be derived at least in part from immune cells. The most predominant metabolic subnetworks contained proteins involved in carbohydrate/alcohol and protein metabolism, with a smaller cluster functioning in lipid metabolism (Fig. 3c; Supplementary Fig. 4). Detailed examination revealed that the molecular function of these proteins is mainly enzymatic, with a remarkable enrichment in glycolytic enzymes as each step of the reaction was represented in the core urinary proteome together with several enzymes involved in pentose phosphate pathway (Fig. 3e; Supplementary Fig. 5). In addition, many enzymes required for the subsequent amino acid biogenesis from the intermediary metabolites were identified in the core urinary EV proteome (Fig. 3e; Supplementary Fig. 5).

Taken together, our functional data mining of core urinary EV proteins showed an enrichment for EV biogenesis, metabolism and immune-related processes that are consistently identified in time and within all donors. These processes are frequently deregulated in diseases, underlining the potential for the use of urinary EVs for the detection of disease.

### Gender-distinct patterns are detected in urinary EVs

**Figure 4:**
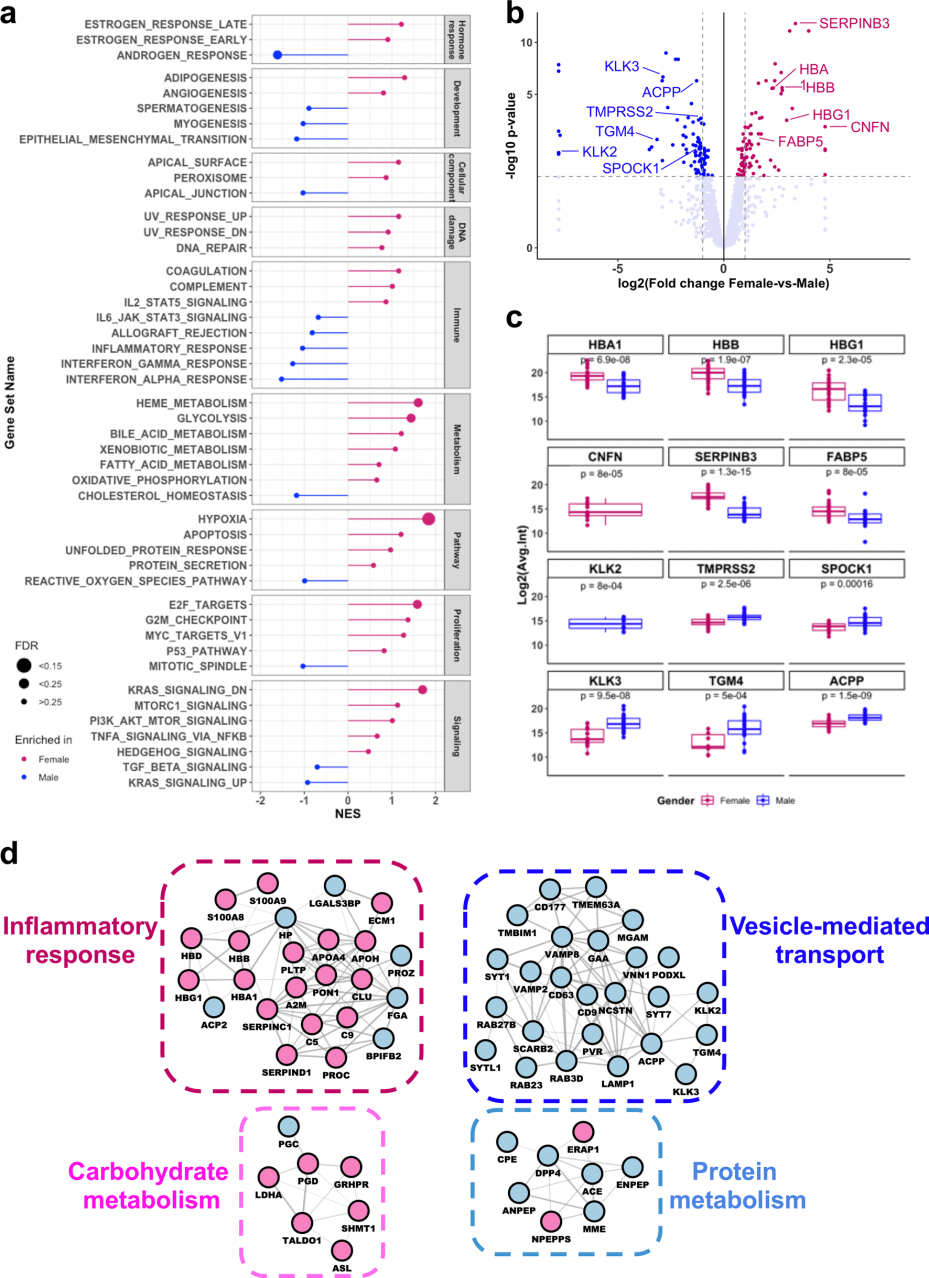
Gender-based differences in the urinary EV proteome. (**a**) Gene set enrichment analysis (GSEA, hallmarks) of the differential analysis between female- and male-derived urinary EVs was performed by ranking proteins based on the sign of their fold change and p-value, with proteins significantly overexpressed in females at the top of the list. Gene sets enriched in females are marked pink, and gene sets enriched in males are marked blue. The size of the dot reflects the significance of the enrichment (false discovery rate). (**b**) Volcano plot of the urinary EV protein expression levels between female and male samples. Colored dots indicate the 172 proteins that were significantly different between female or male samples. A total of 89 proteins were upregulated in males (left side, in blue), whereas 83 proteins were overexpressed in females (right side, pink). Proteins-of-interest that are shown in Fig. 4C are annotated in the plot. (**c**) Expression levels of selected female- (upper 2 panels) and male-specific (lower 2 panels) urinary EV proteins, showing an enrichment of gender-specific proteins in the expected samples. (**d**) Protein interaction networks enriched in female (left) and male (right) urinary EVs, predicted by clusterONE^31^.

Besides intra- and inter-individual variation, we also investigated whether gender differences can be detected in the urinary EV proteome. A complete list of differentially expressed proteins in females and males are provided in Supplementary Table 1. Comparison of female- versus male-derived urinary EV proteomes by Gene Set Enrichment Analysis (GSEA) showed that the androgen response pathway and spermatogenesis are enriched in males, whereas females show increased estrogen response processes (Fig. 4a). Moreover, blood- and oxygen-related pathways such as hypoxia, coagulation, angiogenesis and heme metabolism were enriched in females compared to males (Fig. 4a), further suggesting that reproductive system-based differences are detectable in the urinary EV proteome.

Further inspection of the proteins underlying these signatures showed that multiple hemoglobin subunits were significantly increased in females when compared to males (Fig. 4b,c; Supplementary Fig. 6a); whereas in males, prostate-associated protein KLK2 was present only in male-derived urinary EVs and prostate-implicated proteins such as SPOCK1 and TMPRSS were upregulated in males (Fig. 4b,c; Supplementary Fig. 6a). Furthermore, we also inspected 3 male specific and 3 female specific proteins from the Human Protein Atlas (http://www.proteinatlas.org). All three prostate-secreted proteins (KLK3/PSA, TGM4, ACPP) were significantly increased in male-derived urinary EVs; whereas vagina/cervix-associated proteins SERPINB3 and FABP5 were significantly increased in females, and CNFN uniquely expressed in female urinary EVs (Fig. 4b,c; Supplementary Fig. 6a).

Finally, network visualization of the gender-enriched proteins revealed an inter-connected network mostly within gender, and to a lesser degree between genders (Supplementary Fig. 6b). Analysis of individual protein clusters showed enrichment of immune-related processes and carbohydrate metabolism in female urinary EVs; while enzymes involved in protein metabolism were identified in male-derived urinary EVs (Fig. 4d). Interestingly, in addition to prostate-secreted proteins (ACPP, TGM4, KLK2, KLK3/PSA), several vesicle-related proteins such as RABs and VAMPs, as well as tetraspanins CD9 and CD63 were significantly increased in male urinary EVs, underlining the secretory function of the prostate gland in males (Fig. 4d).

Together this analysis demonstrates that gender-specific proteins and functions can be detected in the urinary EV proteome.

## Discussion

Characterization of the fluctuations in the normal urinary EV proteome provides crucial information for biomarker research. We demonstrate that the majority of the urinary EV proteins are stable in time, and shared between different individuals. The core urinary EV protein networks are involved in EV-related functions, metabolism and immunity, and gender-enriched processes are linked to hormonal and reproductive functions. Our results underscore the value of urinary EV proteins as promising source for biomarker discovery.

A reproducible and clinically-applicable EV isolation method is essential to investigate urinary EV-based functions and biomarkers. Minor non-EV protein contaminants are not a concern as long as these do not obscure the analysis of the EV proteome. The VN96 peptide-based EV isolation provides such a method. It interacts with heat-shock proteins (HSPs) exposed on vesicle surface^20^. Being a charged peptide, it may co-isolate some non-EV associated proteins and nucleic acids. We previously showed that EVs isolated using Vn96-based affinity capture are highly similar to those isolated by ultracentrifugation^21,22^. Comparison to EV fractions isolated using either size focusing (i.e. size exclusion chromatography) or affinity pull down (i.e. using anti-tetraspanin immunobeads) remain to be done. In view of the high affinity for HSPs to pull-down EVs, it is possible that Vn96 introduces a bias for isolating specific EV populations under certain circumstances. For example, HSPs are known to be upregulated in cancer^39^, and therefore the Vn96 method may be advantageous to enrich for cancer EVs. However, in our benchmark study^22^, we did not see differential enrichment of HSPs in the cancer EVs isolated by the VN96 method as compared to ultracentrifugation.

The proteome of full urine may be affected by many factors that can be induced by differences between individuals’ lifestyle such as hydration status, diet, exercise, age, gender and environmental factors among others^10^. In contrast to the full urinary proteome that is quite variable^7–9,11,12^, the urinary EV proteome may be highly stable due to the protection against degradation provided by the lipid bilayer^3^. Previously, two small-scale studies also described low level of variation of the EV proteome, though the depth of these studies was limited (ranging between 500 and 1000 proteins)^13,14^. The high intra- and inter-individual stability of the urinary EV proteome in our in-depth study demonstrates that urinary EV proteins are highly suitable for biomarker studies.

Enriched biological functions in urinary EVs included an EV-biogenesis-linked protein cluster containing ESCRT components, multivesicular body proteins as well as other known EV-markers. These proteins were highly connected with signaling proteins such as signaling transducer G protein subunits and RAB proteins. This may indicate the presence of activated kinases within urinary EVs. That phosphorylated proteins are present in EVs has previously been demonstrated in different in vitro studies^40,41^ and in human plasma^42^*.* Moreover*,* phosphoproteome analyses have verified their activation status also in the urinary EVs^43,44^. Activated kinases within EVs were previously shown to have a functional role in vitro where they can influence the behaviors of recipient cells such as altering their hypoxia status^45^ and metastatic potential^46,47^. Whether or how these signaling proteins have a functional role within the urinary EVs remains unclear.

A remarkable enrichment in glycolytic enzymes was present in urinary EVs with almost 50% of the members of the glycolysis pathway present in the core urinary EV proteome. The presence of a metabolic cluster was previously reported in urinary EVs, mainly focused on TCA cycle and respiratory chain proteins^48,49^. Of note, EV biogenesis-associated and metabolic, especially glycolytic proteins were recently suggested to belong to two distinct EV subpopulations, exosomes and exomeres, respectively^50,51^; suggesting that our urinary EV samples might contain different types of vesicles. Nevertheless, a metabolic, especially glycolytic function appears to be enriched in the urinary EV proteome. The glycolytic pathway is known to be a major driver of immune cell function^52^, which is consistent with the inflammatory protein network in the core urinary EV proteome. This is in line with previous research that suggested a role for urinary EVs in host defense in the urinary tract^53^. Although a part of this immune signature might be derived from immune cells; regardless of origin, detection of inflammatory markers in the urinary EV proteome might provide an opportunity for disease monitoring, or immune-based therapy response monitoring.

Recently, we demonstrated the power of DIA-MS to generate robust, sensitive and reproducible data across eleven different laboratories in nine countries on seven consecutive days in a 24/7 operation mode^17^. This mass spectrometry approach will be highly suitable for future large-scale quantitative proteomics to study the urinary EV proteome under a range of conditions and perturbations, and this approach may also provide a platform for diagnostic applications.

In conclusion, the majority of the urinary EV proteome is stable in time, as well as between different individuals. Therefore, the urinary EV proteome represents an attractive liquid biopsy to identify deregulated proteins in disease and for diagnostic/prognostic applications. These applications may not be limited to diseases of proximal organs such as prostate or bladder cancer, but may also include early detection of distant diseases such as colorectal, lung or breast cancer^6,54,55^.

## Supplementary Information

Supplementary Information.

## Data Availability

The mass spectrometry proteomics data have been deposited to the ProteomeXchange Consortium via the PRIDE^25,26^ partner repository with the dataset identifier PXD022983.
