# Supplementary material for: Longitudinal stability of urinary extracellular vesicle protein patterns within and between individuals
Source: Sci Rep. 2021 Aug 2;11:15629. doi: 10.1038/s41598-021-95082-8 (PMC8329217; doi:10.1038/s41598-021-95082-8)
Supplement: Supplementary file 1 — Supplementary Information. [file 41598_2021_95082_MOESM1_ESM.pdf]

SUPPLEMENTAL INFORMATION LEGENDS

<u>Supplementary Figure 1:</u> Overview of the whole urinary EV proteome including the excluded 5 samples

Total number of proteins identified (upper panel) and distribution of normalized protein intensities (lower panel) for all measured samples (n = 72). Samples excluded from further analysis based on the low protein count (n = 5) are shown with red border.

<u>Supplementary Figure 2:</u> Expression levels of most stable and variable urinary EV proteins

S2a. Expression levels of selected ExoCarta [38] proteins per individual, detected in the top 10% most stable urinary EV proteome.

S2b. Expression levels of top 15 most stable urinary EV proteins per individual.

S2c. Expression levels of top 15 most variable urinary EV proteins per individual.

<u>Supplementary Figure 3: Consistency of the urinary EV proteome within and between individuals</u>

Percentage of proteins identified in all individuals (core proteome, 39.3%), in more than 1 individual (total ~90%), and unique proteins per person (combined 10%)

<u>Supplementary Figure 4:</u> Protein interaction network of the core urinary EV proteome

The protein interaction network of the core urinary EV proteome (516 proteins) was constructed using STRING [29] and was exported to Cytoscape [30] for further analysis. Protein clusters were identified using ClusterONE [31] and were annotated for biological function using BINGO [32]. When multiple biological functions were available for a protein, the most significant one was annotated on the network. When no function could be annotated, a manual search was conducted. Identified main pathways are clustered by a thick line and sub-networks are annotated within each pathway.

<u>Supplementary Figure 5:</u> Metabolic pathways enriched in the urinary EV proteome

Proteins present in the core urinary EV proteome are highlighted in green in glycolysis, pentose-phosphate and amino acid synthesis pathways, adapted from KEGG pathways [56].

<u>Supplementary Figure 6:</u> Gender-related differences in the urinary EV proteome

S6a. Expression of female- (upper 2 panels) and male-specific (lower 2 panels) urinary EV proteins per person per individual.

S6b. Complete network of differentially expressed proteins ($p < 0.05$) in female (pink) and male (blue) urinary EVs.

<u>Supplementary Table 1:</u> Differentially expressed urinary EV proteins between females and males

Supplementary Figure 1

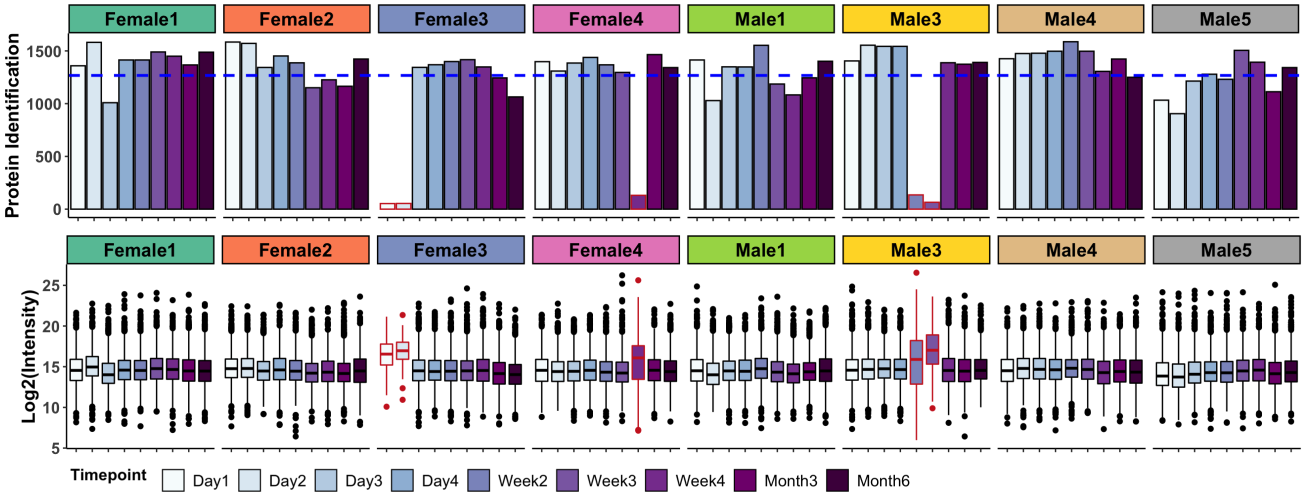

Supplementary Figure 2

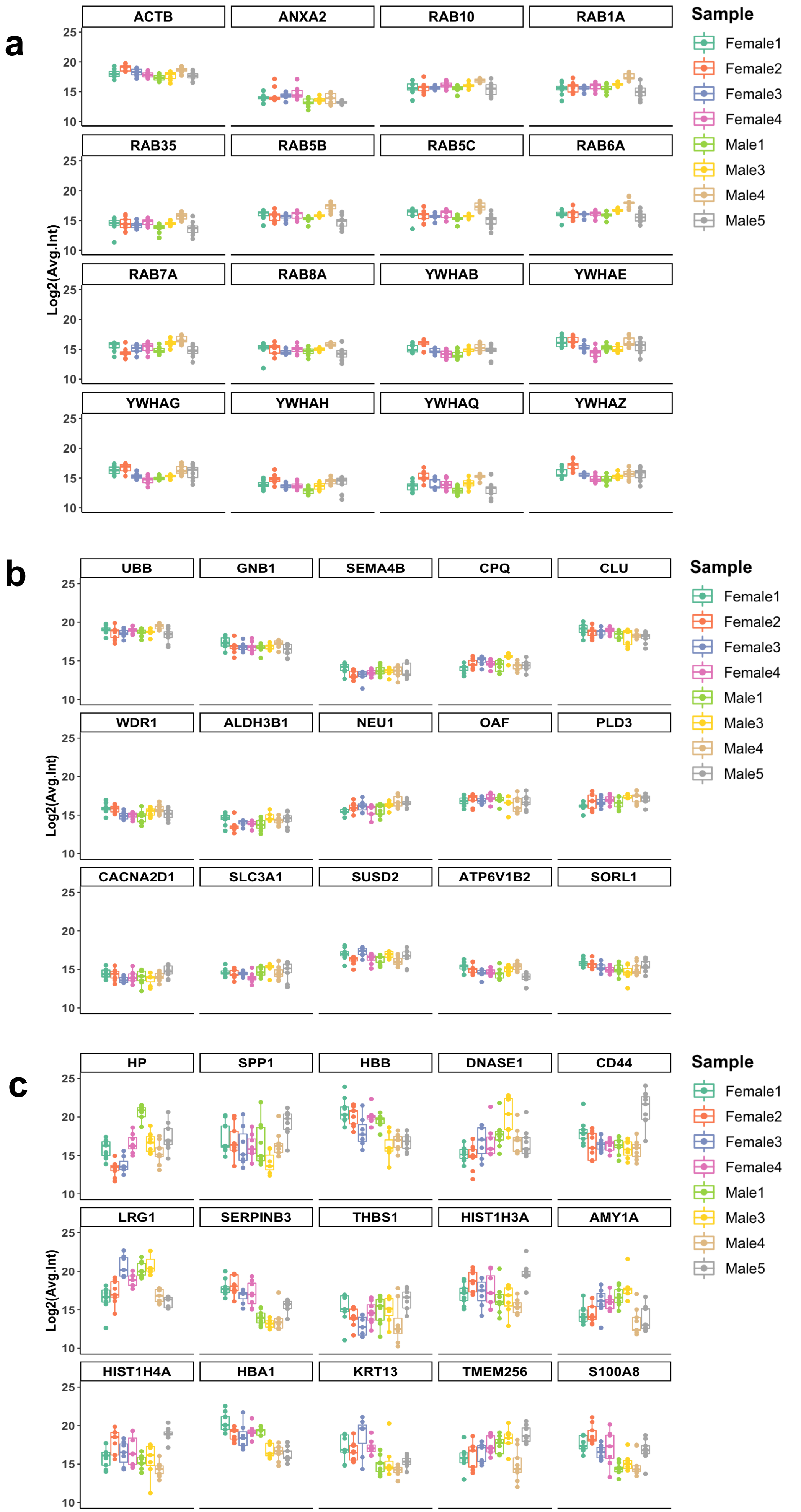

Supplementary Figure 3

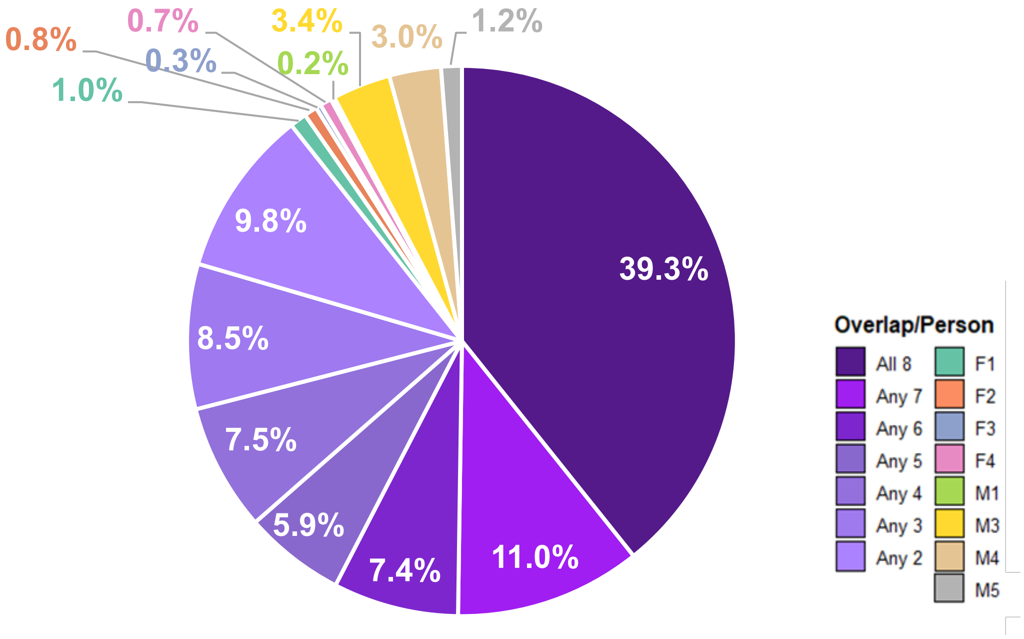

Supplementary Figure 4

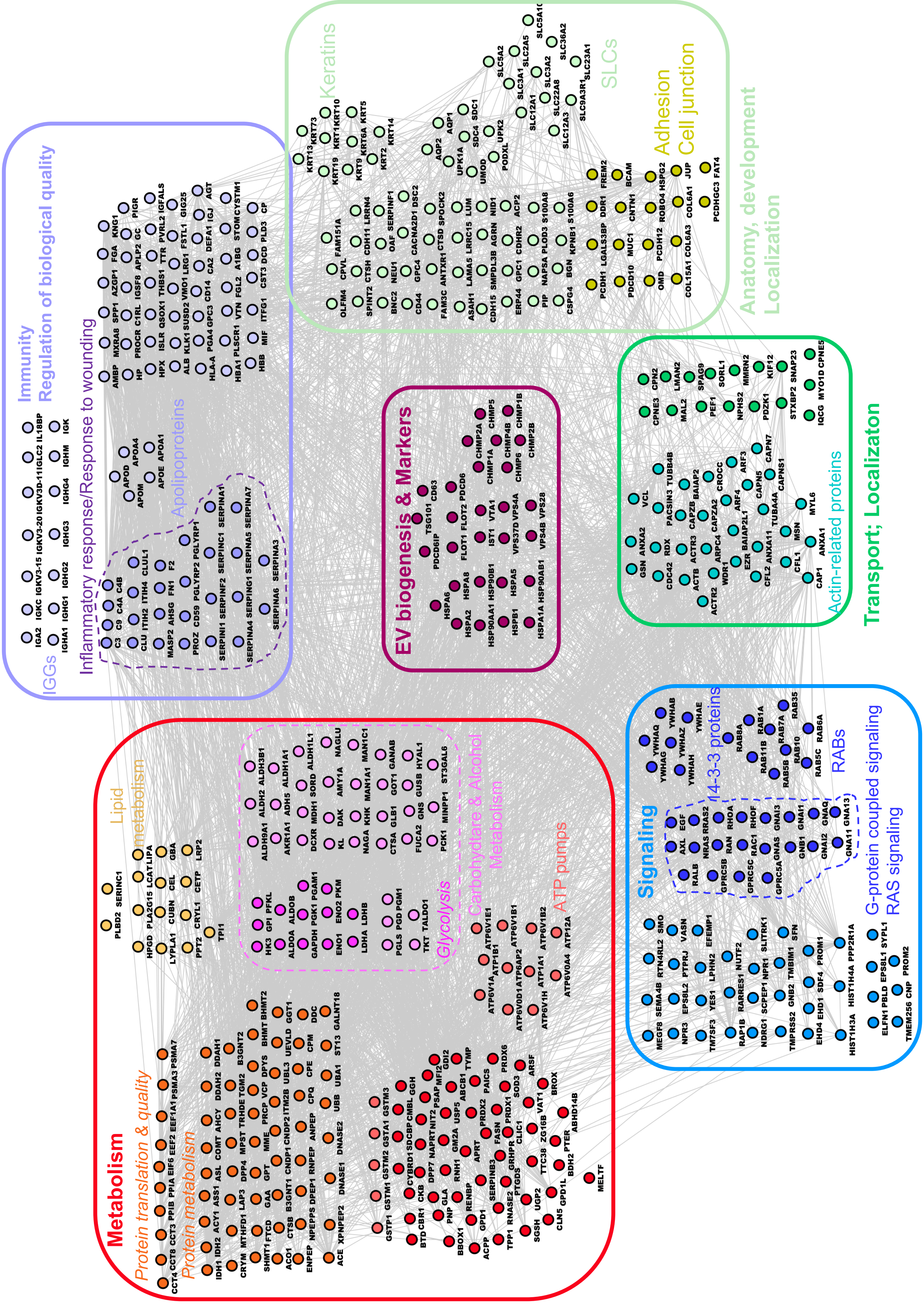

Supplementary Figure 5

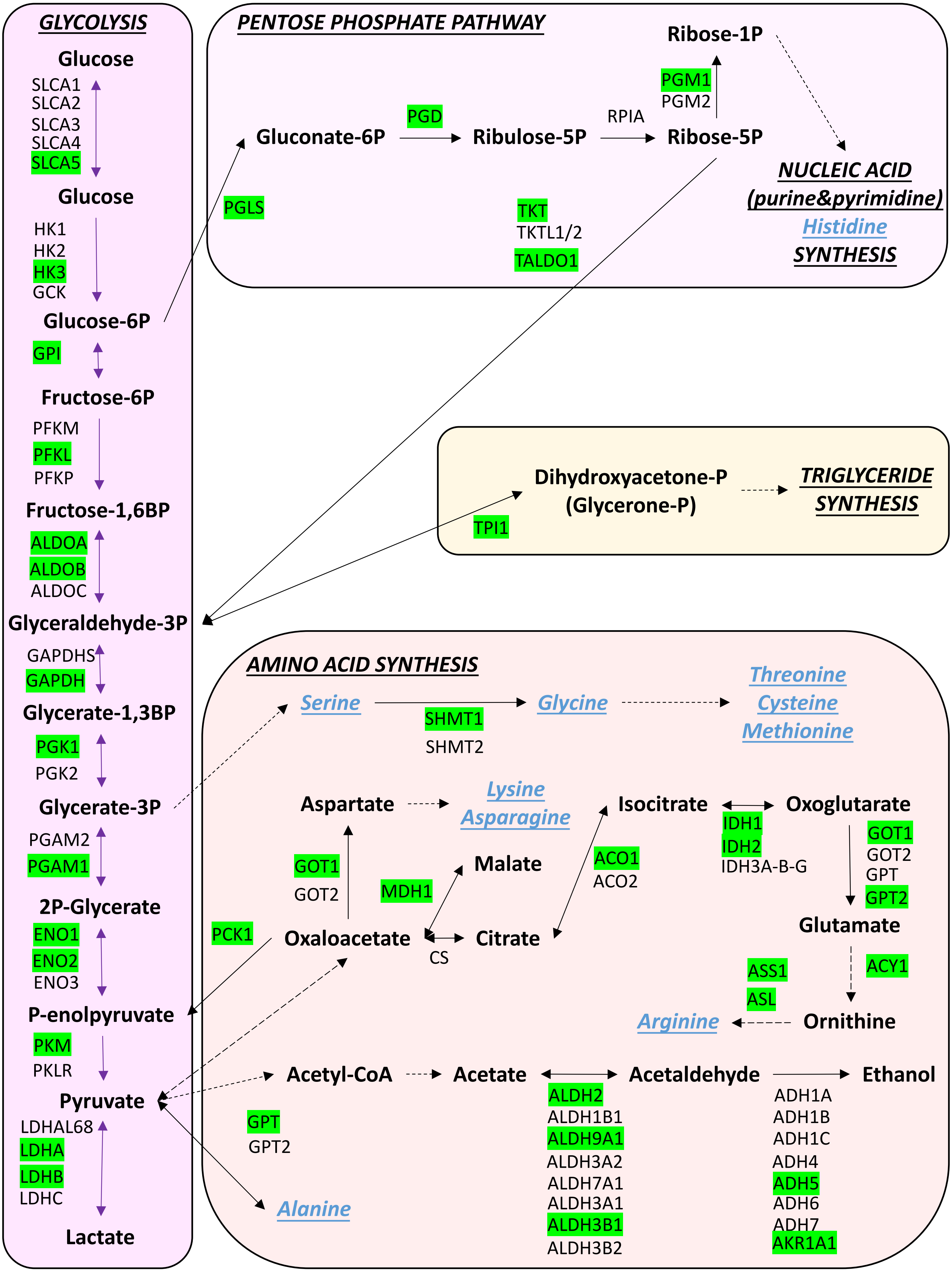

Supplementary Figure 6

a

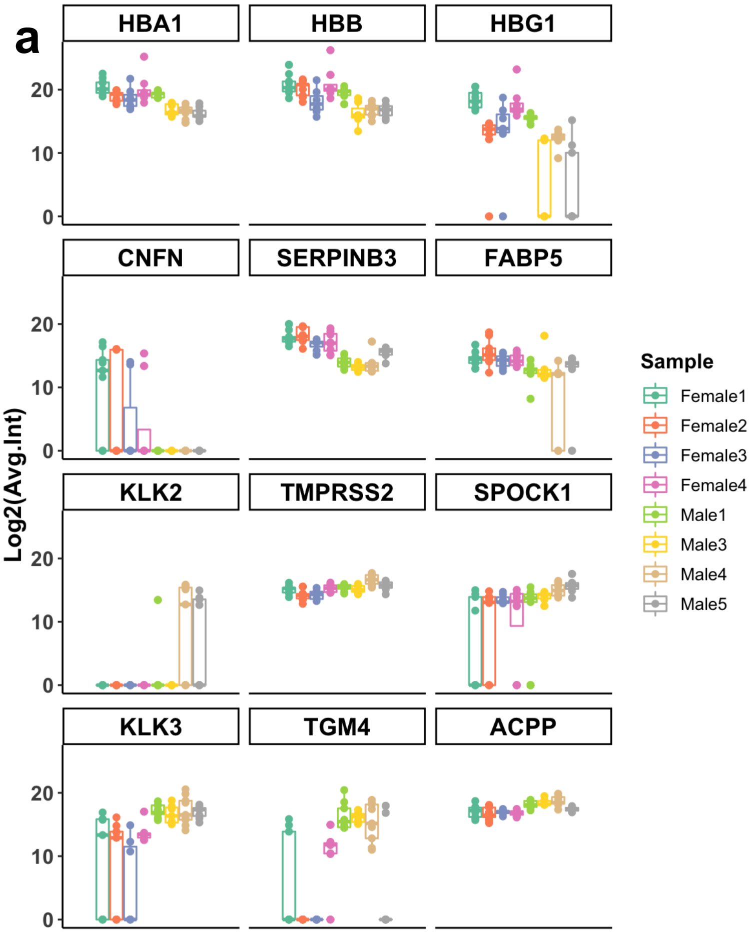

b

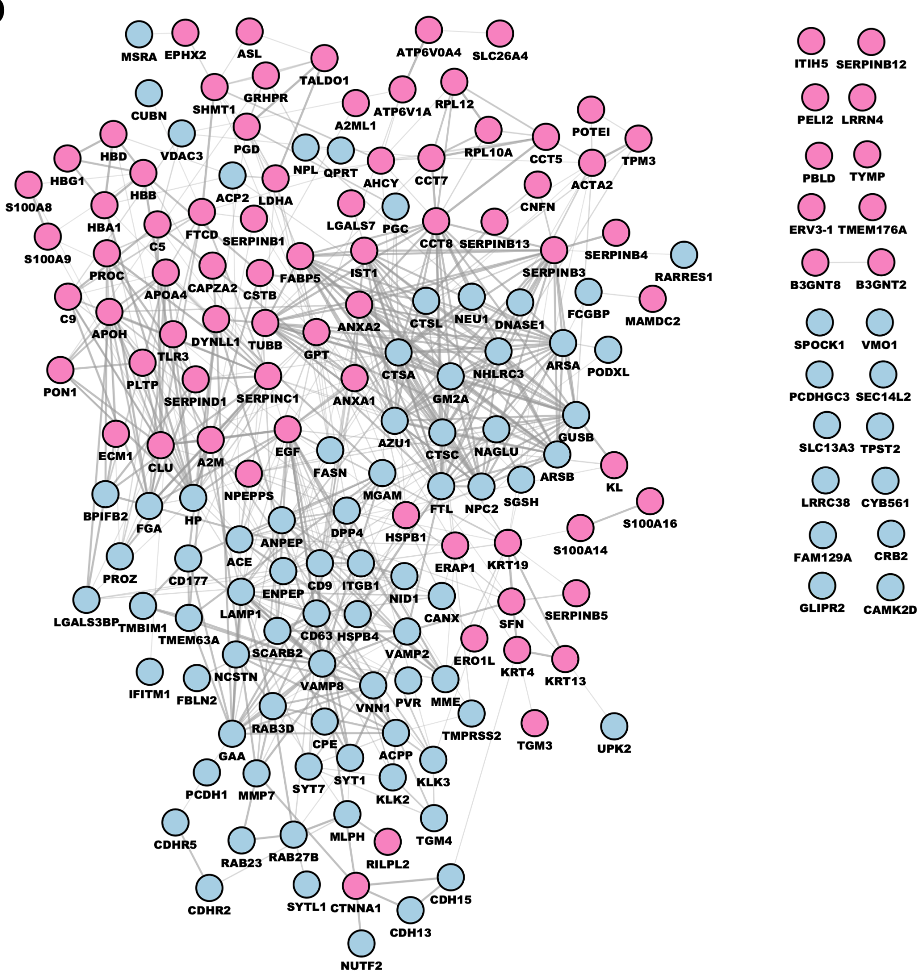

Supplementary Table 1: Differentially expressed urinary EV proteins between females and males

First.Accession	Gene.Name	Fold.Change. Female-vs-Male	Adjusted.p.value. BenjaminiHochberg
Q8IYJ3	SYTL1	-10000.00	3.27E-08
Q9BZQ8	FAM129A	-10000.00	1.36E-07
O43581	SYT7	-10000.00	0.001245702
P20151	KLK2	-10000.00	0.009181041
P21579	SYT1	-10000.00	0.009247534
Q9BV36	MLPH	-10000.00	0.010295001
P98095	FBLN2	-211.86	0.001891316
Q13557	CAMK2D	-11.42	0.007047928
O76054	SEC14L2	-10.60	0.005691239
P49221	TGM4	-8.91	0.0028655
Q9Y6R7	FCGBP	-7.51	9.26E-07
P20160	AZU1	-7.46	0.01700992
P07288	KLK3	-7.33	4.47E-07
O00194	RAB27B	-6.64	1.90E-09
P00738	HP	-6.26	7.16E-05
Q8N6Q3	CD177	-5.31	0.004734369
O95716	RAB3D	-4.84	9.28E-09
P49447	CYB561	-4.59	0.000352019
P55291	CDH15	-4.45	9.28E-09
P24855	DNASE1	-3.78	0.004734369
Q15274	QPRT	-3.78	0.001891316
Q9BXD5	NPL	-3.71	0.019230034
Q5VT99	LRRC38	-3.57	0.000778741
Q9H4G4	GLIPR2	-3.46	0.002264586
Q8N4F0	BPIFB2	-3.43	0.00694433
P05556	ITGB1	-3.33	0.009550765
P16870	CPE	-3.21	0.000268542
P02489	CRYAA	-2.92	0.024611145
Q9HBB8	CDHR5	-2.89	4.08E-05
P02792	FTL	-2.73	0.000457086
Q7Z5L0	VMO1	-2.72	0.001680906
P55290	CDH13	-2.72	0.004734369
P49788	RARRES1	-2.61	0.018576086
Q5JS37	NHLRC3	-2.55	0.008983708
Q92542	NCSTN	-2.52	0.031959725
P27824-2	CANX	-2.52	0.008488549
Q08380	LGALS3BP	-2.51	0.005043204
P08962	CD63	-2.50	0.011722787
P02671	FGA	-2.50	0.011220947
Q5IJ48	CRB2	-2.46	0.040631353
P15309	ACPP	-2.44	9.26E-07
Q08174	PCDH1	-2.35	0.003543264
P20142	PGC	-2.34	0.033242112
P11279	LAMP1	-2.33	0.002422723
P15144	ANPEP	-2.28	0.000353402
Q9Y277	VDAC3	-2.27	0.038401496
O43451	MGAM	-2.26	0.0028655
P51688	SGSH	-2.24	0.006619839
P49327	FASN	-2.22	0.005691239
P22891	PROZ	-2.22	0.006767551
O60704	TPST2	-2.22	0.018255027
P54802	NAGLU	-2.22	0.011601942
Q969X1	TMBIM1	-2.17	0.003875578
Q07075	ENPEP	-2.16	0.016061143
P27487	DPP4	-2.16	0.004221379
Q08629	SPOCK1	-2.15	0.006513971
P11117	ACP2	-2.13	0.009489716
P14543	NID1	-2.11	0.005668521
O15393	TMPRSS2	-2.11	0.000289874
P17900	GM2A	-2.10	0.045218326
P12821	ACE	-2.07	0.000518731
O95497	VNN1	-2.06	0.015677504
Q9ULC3	RAB23	-2.05	0.023798828
P10253	GAA	-2.05	0.047863845
P15289	ARSA	-2.05	0.014648644
P08236	GUSB	-2.05	0.015711295
Q9UJ68	MSRA	-2.03	0.027962461

P21926	CD9	-2.01	0.045280871
P61970	NUTF2	-2.00	0.046173676
P09237	MMP7	-1.98	0.019677975
Q8WWT9	SLC13A3	-1.96	0.033164556
Q14108	SCARB2	-1.96	0.016668974
Q9UN70	PCDHGC3	-1.93	0.000577093
P13164	IFITM1	-1.92	0.047863845
O00592	PODXL	-1.92	0.041184276
P15848	ARSB	-1.92	0.016061143
P10619	CTSA	-1.91	0.010385823
O00526	UPK2	-1.91	0.033735205
P07711	CTSL	-1.89	0.006413785
Q9BYE9	CDHR2	-1.87	0.007585278
Q9BV40	VAMP8	-1.86	0.008488549
P53634	CTSC	-1.86	0.022076964
P15151	PVR	-1.85	0.020659389
P61916	NPC2	-1.83	0.013462747
P08473	MME	-1.79	0.011722787
O94886	TMEM63A	-1.70	0.013000087
O60494	CUBN	-1.68	0.045280871
P63027	VAMP2	-1.67	0.007592381
Q99519	NEU1	-1.46	0.046928875
Q9UEF7	KL	1.57	0.043407228
P10909	CLU	1.59	0.006802172
Q9NY97	B3GNT2	1.64	0.045280871
P23526	AHCY	1.65	0.031959725
Q9NZ08	ERAP1	1.72	0.006767551
P19971	TYMP	1.76	0.035246341
P47755	CAPZA2	1.76	0.021881672
P53990-2	IST1	1.76	0.046173676
A0A0C4DH41	IGHV4-61	1.78	0.046173676
P34896	SHMT1	1.79	0.013632327
P08727	KRT19	1.79	0.024401019
Q9UBQ7	GRHPR	1.80	0.010798425
Q7Z7M8	B3GNT8	1.80	0.023139553
P01133	EGF	1.81	0.006513971
P48643	CCT5	1.81	0.007592381
P50990	CCT8	1.82	0.013329356
Q9HAT8	PELI2	1.83	0.035387948
Q99832	CCT7	1.84	0.019648215
P07355	ANXA2	1.88	0.00166712
P55058	PLTP	1.89	0.003463029
P02748	C9	1.91	0.045280871
P01008	SERPINC1	1.95	0.026362329
P04080	CSTB	1.96	0.016668974
Q9HBG4	ATP6V0A4	1.96	0.02926863
P02749	APOH	1.98	0.03719539
P04792	HSPB1	1.99	0.024028359
Q8WUT4	LRRN4	1.99	0.00254447
O95954	FTCD	2.00	0.024401019
P00338	LDHA	2.01	0.000806757
P38606	ATP6V1A	2.02	0.013329356
P04424	ASL	2.03	0.005620676
P04070	PROC	2.03	0.021820764
Q86UX2	ITIH5	2.09	0.004454783
P30039	PBLD	2.10	0.00694433
O15455	TLR3	2.10	0.013462747
P07437	TUBB	2.12	0.012956735
Q7Z304	MAMDC2	2.14	0.004734369
P24298	GPT	2.21	0.000848288
P62906	RPL10A	2.29	0.027227607
Q9HCY8	S100A14	2.29	0.003286095
P06727	APOA4	2.32	0.003876384
P30050	RPL12	2.34	0.020796673
P34913	EPHX2	2.36	0.006513971
P37837	TALDO1	2.38	0.001639332
P55786	NPEPPS	2.40	0.000667665
Q969X0	RILPL2	2.43	0.024611145
P05546	SERPIND1	2.44	0.001639332
Q96P63-2	SERPINB12	2.48	0.004693205
P52209	PGD	2.50	0.0001624
P31947	SFN	2.54	0.013462747
O43511	SLC26A4	2.63	0.041652273

P01031	C5	2.64	0.011793199
P30740	SERPINB1	2.84	0.000142067
P01023	A2M	2.94	0.018118446
Q08188	TGM3	3.10	0.004347306
P35221	CTNNA1	3.10	1.47E-06
Q01469	FABP5	3.11	0.001639332
P47929	LGALS7	3.22	0.026742036
P27169	PON1	3.22	0.000289874
Q96FQ6	S100A16	3.39	0.00166712
P19013	KRT4	3.47	0.000264996
P63167	DYNLL1	3.70	0.046928875
P04083	ANXA1	3.97	9.26E-07
P0CG38	POTEI	4.62	0.01650879
P05109	S100A8	4.84	3.69E-06
P69905	HBA1	5.01	3.48E-06
P13646	KRT13	5.28	9.26E-07
Q16610	ECM1	5.29	0.026681074
P36952	SERPINB5	5.35	2.69E-08
P62736	ACTA2	5.99	0.033242112
P48594	SERPINB4	6.49	9.01E-06
P06702	S100A9	6.52	1.81E-07
P68871	HBB	6.71	3.28E-06
P02042	HBD	6.76	5.15E-06
P69891	HBG1	7.80	0.000367605
A8K2U0	A2ML1	8.61	2.62E-12
Q96HE7	ERO1A	9.39	8.17E-05
P29508	SERPINB3	10.39	1.94E-13
Q9UIV8	SERPINB13	16.03	2.62E-12
Q9BYD5	CNFN	10000.00	0.000770822
Q96HP8	TMEM176A	10000.00	0.006872338
P06753-2	TPM3	10000.00	0.007558296
Q14264	ERV3-1	10000.00	0.04460078
